# Clinical expressions, characteristics and treatments of confirmed COVID-19 in nursing home residents: a systematic review

**DOI:** 10.1186/s12877-023-03826-0

**Published:** 2023-02-17

**Authors:** Anita Nilsen, Bjørn Lichtwarck, Siren Eriksen, Anne Marie Mork Rokstad

**Affiliations:** 1 Faculty of Health Sciences and Social Care, Molde University College, Molde, Norway; 2 The Research Centre for Age-Related Functional Decline and Disease, Innlandet Hospital Trust, Ottestad, Norway; 3 Norwegian National Centre for Ageing and Health, Vestfold Hospital Trust, Tønsberg, Norway; 4 Lovisenberg Diaconal University College, Oslo, Norway

**Keywords:** COVID-19, Nursing home, Older adult, Symptom, Comorbidity, Mortality

## Abstract

**Background:**

The coronavirus 2019 (COVID-19) pandemic has led to a high rate of infections, frequent outbreaks, and high mortality rates in nursing homes (NH) worldwide. To protect and improve the treatment and care of the vulnerable NH population, it is pivotal to systematise and synthesise data from cases of COVID-19 among NH residents. In our systematic review, we therefore aimed to describe the clinical expressions, characteristics, and treatments of NH residents confirmed to have COVID-19.

**Methods:**

We conducted two comprehensive literature searches in several electronic databases: (1) PubMed, (2) CINAHL, (3) AgeLine, (4) Embase, and (5) PsycINFO in April and July 2021. Of the 438 articles screened, 19 were included in our sample, and we used the Newcastle–Ottawa Assessment Scale to assess the quality of the reported studies. A weighted mean (*M*_weighted_), was calculated to account for the large variation in sample sizes of the studies, and due to heterogeneity among the studies, we report our findings in a narrative synthesis.

**Results:**

According to the mean weights (*M*_weighted_), common symptoms and signs in NH residents confirmed to have COVID-19 were fever (53.7%), cough (56.5%), hypoxia (32.3%), and delirium or confusion (31.2%). Common comorbidities were hypertension (78.6%), dementia or cognitive impairment (55.3%), and cardiovascular diseases (52.0%). Six studies presented data concerning medical and pharmacological treatments, such as inhalers, oxygen supplementation, anticoagulation, and parenteral/enteral fluids or nutrition. The treatments were used to improve outcomes, as part of palliative care, or as end-of-life treatment. Transfers to hospital for NH residents with confirmed COVID-19 were reported in six of the included studies, and the rate of hospital transfers ranged from 6.9% to 50% in this population. In the 17 studies reporting mortality, 40.2% of the NH residents died during the studies’ observation periods.

**Conclusions:**

Our systematic review allowed us to summarise important clinical findings about COVID-19 among NH residents and to identify the population’s risk factors for serious illness and death caused by the disease. However, the treatment and care of NH residents with severe COVID-19 warrant further investigation.

## Background

Following the first known outbreak of an unknown virus in December 2019, the virus was identified in January 2020 as a novel coronavirus, namely severe acute respiratory syndrome (SARS) coronavirus 2 (SARS-CoV-2), and in March 2020, the World Health Organization (WHO) declared the viral outbreak to be a pandemic [1]. SARS-CoV-2 has genetic similarities to the SARS and Middle East respiratory syndrome viruses, which also belong to the coronavirus family. SARS-CoV-2 causes coronavirus disease (COVID-19), the effects of which range from mild symptoms to serious illness and even death [2]. Common signs and symptoms of COVID-19 include not only fever, cough and malaise, but also sore throat, nausea, and vomiting. Loss of taste and/or smell may also occur. The primary modes of COVID-19’s transmission are close contact with infected others, exposure to droplets containing SARS-CoV-2, and contact with surfaces contaminated by the virus [2]. As of August 2022, the pandemic remains ongoing, the virus has undergone various mutations, and the WHO has reported a worldwide total of 593,269,262 confirmed cases and 6,446,547 deaths [3]. Major resources have been invested in developing safe and effective vaccines against COVID-19, the first of which were introduced for use in December 2020. Although the approved vaccines are intended to provide protection against serious illness, booster doses have been necessary to maintain vaccine efficacy [4, 5]. According to the WHO, 12,409,086,286 vaccine doses against COVID-19 have been administered worldwide as of August 2022 [3].

Nursing homes (NH), as institutions primarily serving older adults who need 24-h health and care services, host a vulnerable, clinically complex population characterised by both physical and mental multimorbidity and decreased function in activities of daily living [6–8]. Studies have revealed that NH residents are mostly adults at least 80 years old. More than 80% have dementia or cognitive impairment, and the number of both mental and physical diagnoses is likely to be three or more per NH resident [7–9]. Individuals who are more than 60 years old and who have a least one underlying health condition are at higher risk of severe COVID-19 than their younger counterparts. Beyond that, the risk of death from COVID-19 increases with age, and people 80 years old or older thus have the greatest risk of mortality [10, 11].

The COVID-19 pandemic hit NHs worldwide especially hard and included frequent outbreaks and high mortality rates [12, 13]. To confine the transmission of the virus and outbreaks of COVID-19 in NHs, actions such as visitation restrictions, cohorting, and isolation have been implemented. As a consequence, the additional strains associated with such pandemic precautions have probably caused major negative consequences for the mental and physical health of many NH residents [6].

NH residents are particularly vulnerable to outbreaks of infectious disease compared with other populations given their combination of physical and mental multimorbidity, old age, immunosenescence, low functioning in activities of daily living (ADL), increased frailty, high dependence on nursing staff, and the fact that they cohabitate in grouped living environments [7, 8, 14]. Among NH residents, the atypical presentation of infections in the form of delirium, falls, and the absence of fever is often observed [14–16]. Against that background, diagnosing infectious diseases in the population can be challenging [14, 15].

Only a limited number of systematic reviews pertaining to the topic of NH residents and COVID-19 have been published. Worth mentioning is the study by Hashan et al. [17] who conducted a systematic review and meta-analysis with a focus on epidemiology and clinical features of COVID-19 outbreaks in aged care facilities. In response, to safeguard and improve treatment and care for such a vulnerable population both during the current pandemic and in anticipated future epidemics and pandemics, it is pivotal to systematise and synthesise data regarding cases of COVID-19 among NH residents. To our knowledge, a lack of systematic reviews describing medical and pharmacological treatments of NH residents with confirmed COVID-19 exist. Thus, the aim of our systematic review was to describe clinical expressions, characteristics and treatments of NH residents confirmed to have COVID-19.

## Methods

### Inclusion and exclusion criteria

Our review was conducted in accordance with the Preferred Reporting Items for Systematic reviews and Meta-Analyses (PRISMA) 2020 statement [18].

In our sample, we included all articles reporting primary quantitative studies relevant to the clinical expressions, characteristics and treatments of confirmed cases of COVID-19 among NH residents. Studies written in languages other than English, Norwegian, Danish, or Swedish were excluded.

In this systematic review we use the term “nursing home” as a label for the institutions in the articles included in our study. We acknowledge that different interpretations of the term “nursing home” in the different countries can be found, but in this systematic review the term is used for institutions run by health care professionals serving older adults who need 24-h health and care services. Sanford et al. (2015) offers a definition of NHs that states that NHs are facilities “that provides 24-h functional support and care for persons who require assistance with ADLs and who often have complex health needs and increased vulnerability” [19]. The institutions in the included articles in this systematic review are covered by this definition.

### Search strategy

Two comprehensive searches for literature in different electronic databases were conducted using combined search terms related to “COVID-19” and “nursing home”. The first search for literature, conducted on 22 April 2021, probed the databases PubMed, CINAHL, and AgeLine, whereas the second, conducted on 5 July 2021, probed those three databases in addition to the EMBASE and PsycINFO databases. The search terms used in the second search differed somewhat for the various databases, as presented in the search log in Table 1. For both searches, medical subject headings (MeSH) and a combination of the search fields “Title”, “Abstract”, “Heading word” and “Key word” were used when available to ensure the best possible searches. We also re-examined our searches of the databases and searches of the reference lists of the articles for other eligible literature. In addition, a recommended study identified through manual literature searches was also assessed but excluded due to overlap of data with another study in this review.

**Table 1:** Search log

22 April 2021	
Database	Search terms
PubMed	((COVID-19) AND (nursing home) AND (((nursing[MeSH Terms] OR (health care[Title/Abstract] OR (health caring[Title/Abstract] Filters: English
CINAHL	((COVID-19) AND (nursing home) AND (((nursing[MeSH Terms] OR (health care[Title/Abstract] OR (health caring[Title/Abstract] Filters: English
AgeLine	((COVID-19) AND (nursing home) AND (((nursing[MeSH Terms] OR (health care[Title/Abstract] OR (health caring[Title/Abstract] Filters: English
5 July 2021	
Database	Search terms
PubMed	(((((((long-term care[MeSH Terms]) OR (long-term care[Title/Abstract])) OR (residential care[Title/Abstract])) OR (nursing home[MeSH Terms])) OR (nursing home resident*[Title/Abstract])) AND (((COVID-19) OR (coronavirus)) OR (SARS-CoV-2))) AND ((disease course and care) OR (treatment or therapy))) AND (assessment and measures) Filters: English
CINAHL	COVID-19 or coronavirus or SARS-CoV-2) AND (nursing home or residential care OR long-term care OR nursing home residents) AND (treatment or therapy) OR (care or nursing)
AgeLine	(COVID-19 or coronavirus or SARS-CoV-2) AND (nursing home or residential care OR long term care OR nursing home residents) AND (treatment or therapy) OR (care or nursing)
EMBASE	(COVID-19 or coronavirus or SARS-CoV-2. [Title/Abstract/Heading word/Keyword]) AND (nursing home residents or long-term care or nursing home or residential care). [Title/Abstract/Heading word/Keyword]AND (treatment or therapy) AND (disease course and care). [Title/Abstract/Heading word/Keyword]
PsycINFO	(COVID-19 or coronavirus or SARS-CoV-2. [Title/Abstract]AND (nursing home residents or long-term care or nursing home or residential care). AND (treatment or therapy) [Title/Abstract]

### Screening and selection of studies

After removing duplicate articles from the searches for literature done using various electronic databases, all titles and abstracts from the remaining articles (*n* = 438) were screened. The de-duplication process was conducted by a principal librarian at the Norwegian National Centre for Ageing and Health.

**Fig. 1:**
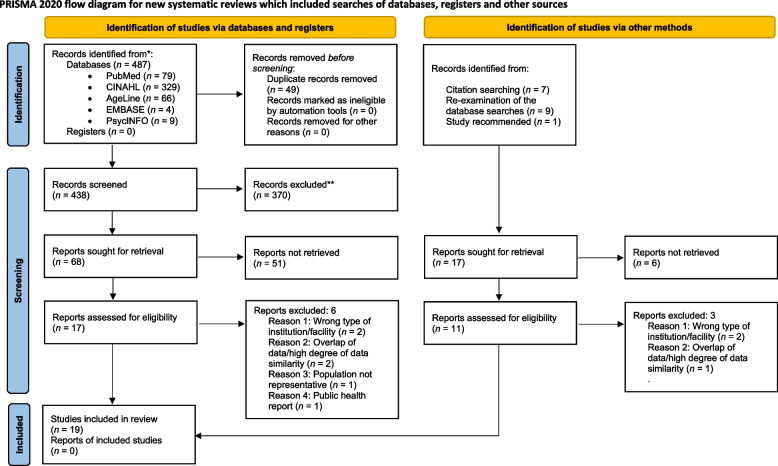
Preferred Reporting Items for Systemic Reviews and Meta-analysis (PRISMA) 2020 flow diagram

In addition, 17 studies identified via other methods were assessed. The flow diagram for the searches for literature (the PRISMA 2020 flow diagram) and screening appears in Fig. 1. After 427 were excluded, 28 eligible articles were read in full. Of them, 9 were excluded for various reasons, as also shown in Fig. 1. The screening and selection of the studies were conducted individually by all researchers, and any disagreements were discussed until consensus was reached.

### Quality assessment and data extraction

To assess quality of the included studies the Newcastle–Ottawa assessment scale (NOS) was used. The studies were assessed according to three perspectives: (1) selection of the population/study group, (2) comparability of the groups, and (3) assessment of outcome [20]. Quality scores were identified by assigning stars for the different categories, and all 19 included studies in this review achieved seven stars or more of nine possible stars according to NOS. Three researchers (AN, BL and AMMR) assessed the quality of the included studies.

A data extraction form was prepared that included study characteristics, including authors, year of publication, country, aim, study design/methods, number of participants, and outcomes. All researchers were involved in the process of data extraction.

Any disagreements in the quality assessment and data extraction phase were discussed by the researchers until a consensus was reached.

Characteristics of the included studies are presented in Table 2.

**Table 2:** Characteristics of the included studies

Authors/year/country	Aim	Study design/methods	Sample	Outcomes
Arons M. M., Hatfield K. M., Reddy S. C., Kimball A., James A., Jacobs J. R. et al. 2020, USA [21]	To assess transmission of SARS-Cov-2 and evaluate the adequacy of symptom-based screening to identify infections in residents	Two serial point-prevalence surveys: 13/3–20 and 19/3–20, using RT-PCR test^a^ A standardised symptom-assessment form was completed by nurses for each resident tested Follow-up period: 13/3–20 to 3/4–20	57 of 89 nursing home residents in a skilled nursing facility tested positive for SARS-CoV-2	Symptoms, signs, specimen sequencing, hospitalisation, and mortality
Atalla E., Zhang R., Shehadeh F., Mylona E. K., Tsikala-Vafea M., Kalagara S. et al. 2021, USA [22]	To report a COVID-19 outbreak in a nursing home focusing on clinical presentation and characteristics associated with mortality	Data were retrospectively collected from residents’ electronic health records COVID-19 confirmed by RT-PCR test Follow-up period: 14/4–20 to 15/6–20	111 of 116 nursing home residents with confirmed COVID-19	Symptoms, signs, comorbidity, laboratory findings, medical treatment, and mortality
Beiting K. J., Huisingh-Scheetz M., Walker J., Graupner J., Martinchek M., Thompson K. et al. 2021, USA [23]	(1) Present a novel clinical management pathway based on a COVID-19 outbreak (2) Describe the demographics, clinical characteristics, course, and outcomes of the population	Single-centre, retrospective, and observational cohort study COVID-19 confirmed by RT-PCR test Follow-up period: 1/3–20 to 31/5–20	172 of 204 nursing home residents in a skilled nursing facility with confirmed COVID-19	Symptoms, signs, comorbidities, clinical management of COVID-19, goals-of-care conversations, hospitalisation, and mortality
Bielza R., Sanz J., Zambrana F., Arias E., Malmierca E., Portillo L. et al. 2021, Spain [24]	To describe the clinical characteristics, 30-day mortality, and associated factors of patients living in nursing homes (NH) with COVID-19	Descriptive, observational retrospective and longitudinal study COVID-19 confirmed by RT-PCR test Follow-up period: 20/3–20 to 1/6–20	630 patients with confirmed COVID-19	Symptoms, signs, comorbidity, medical treatment, hospitalisation, and mortality
Brouns S. H., Brüggemann R., Linkens A. E. M. J. H., Magdelijns, F. J. Joosten H., Heijnen, R. et al. 2020, The Netherlands [25]	To investigate whether the use of oral antithrombotic therapy (OAT) was associated with a lower mortality in NH residents with COVID-19	A retrospective case series COVID-19 confirmed by RT-PCR test Follow-up period: 20/3–20 and 1/5–20	67 of 101 nursing home residents with confirmed COVID-19	Comorbidity, all-cause mortality. Association between age, sex, comorbidity, OAT, and mortality
De Spiegeleer A., Bronselaer A., Teo J. T., Byttebier G., De Tré G., Belmans, L. et al. 2020, Belgium [26]	To explore the association of Angiotensin-converting enzyme inhibitors (ACEis), angiotensin II receptor blockers (ARBs) and/or statins with clinical manifestations in COVID-19-infected older adults residing in nursing homes	Retrospective multicentre cohort study COVID-19 confirmed by RT-PCR test Follow-up period: 1/3–20 to 16/4–20	154 nursing home residents with confirmed COVID-19	Comorbidity, symptoms/no symptoms, and the use of ACEi/ARB and /or statins
Graham, N. S. N., Junghans C., Downes R., Sendall C., Lai H., McKirdy A. et al. 2020, Great Britain [27]	To report results from an outbreak investigation carried out in four UK nursing homes affected by COVID-19	Two point-prevalence surveys were performed one week apart where residents underwent SARS-CoV-2 testing COVID-19 confirmed by RT-PCR test Follow-up period: 1/3–20 to 1/ 5–20	126 of 313 nursing home residents with confirmed COVID-19	Symptoms, signs, comorbidities, SARS-CoV- 2 sequencing, and mortality
Kittang B. R., Hofacker S. V., Solheim S. P., Krüger K., Løland K. K., and Jansen K. 2020, Norway [28]	To identify the COVID-19 infection pathways, course of disease and resident mortality rates	Retrospective observational study COVID-19 confirmed by RT-PCR test Follow-up period: 19/3–20 to 25/4–20	40 nursing home residents with confirmed COVID-19	Symptoms, signs, comorbidity, and mortality
Lally M. A., Tsoukas P., Halladay C. W, O'Neill E., Gravenstein S., and Rudolph J. L. 2021, USA [29]	To evaluate mortality benefit among older persons infected with SARS-CoV-2 who were taking metformin as compared to those who were not	Retrospective cohort study Laboratory confirmation of COVID-19 Follow-up period: 1/3–20 to 13/5–20	775 nursing home residents with confirmed COVID-19	Comorbidity, use of diabetes medication, and mortality
Panagiotou O. A., Kosar C. M., White E. M., Bantis L. E, Yang X., Santostefano C. M. et al. 2021, USA [30]	To identify risk factors for 30-day all-cause mortality among US nursing home residents with COVID-19	Cohort study COVID-19 confirmed by RT-PCR test Follow-up period: 6/3–20 to 15/9–20	5256 nursing home residents with confirmed COVID-19 across 25 states in the United States	Symptoms, signs, comorbidity, sociodemographic characteristics, functional impairment, and mortality
Patel M. C., Chaisson L. H., Borgetti S., Burdsall D., Chugh R. K., Hoff C. R. et al. 2020, USA [31]	(1) To determine the proportion of cases who were symptomatic, presymptomatic, and asymptomatic and (2) incidence of symptoms among those who tested negative	Point prevalence testing 15/3–20 via RT-PCR test Nursing staff interviewed residents and recorded symptoms in residents’ medical charts Follow-up period: 15/3–20 to 14/4–20	35 of 126 nursing home residents in a skilled nursing facility with confirmed SARS-CoV-2	Symptoms, signs, and mortality
Poloni T. E., Carlos A. F, Cairati M., Cutaia C., Medici V., Marelli E. et al. 2020, Italy [32]	To identify the prevalence and prognostic significance of delirium as the sole onset manifestation of COVID-19	Retrospective single-centre study based on review of medical charts COVID-19 confirmed by RT-PCR test Follow-up period: 27/3–20 to 18/4–20	57 of 59 long-term care residents in a dementia special care unit with confirmed COVID-19	Delirium/no delirium at onset COVID-19, comorbidity, laboratory tests, and mortality
Rutten J. J. S., van Loon A. M., van Kooten J., van Buul L. W., Joling K. J., Smalbrugge M. et al. 2020, The Netherlands [33]	To describe the symptomatology, mortality, and risk factors for mortality in nursing home (NH) residents with clinically suspected COVID-19	Prospective cohort study COVID-19 confirmed by RT-PCR test Data were collected via electronic health records Follow-up period: 18/3–20 to 13/5–20	1538 of 4007 nursing home residents with confirmed COVID-19	Symptoms, signs, comorbidity, and mortality
Sacco G., Foucault G., Briere O., and Annweiler C. 2020, France [34]	To describe symptoms and chronological aspects of the diffusion of the SARS-CoV-2 virus in a nursing home	Retrospective cohort study COVID-19 confirmed by RT-PCR test Follow-up period: 17/3–20 to 26/4–20	41 of 87 nursing home residents with confirmed COVID-19	Symptoms, signs, attack rate, and mortality
Shi S. M., Bakaev I., Chen H., Travison T. G., and Berry S. D. 2020, USA [35]	To describe clinical characteristics and risk factors associated with COVID-19 in long-stay nursing home residents	Retrospective cohort study COVID-19 confirmed by RT-PCR test Follow-up period: 16/3–20 to 8/5–20	146 of 389 long-term nursing home residents with confirmed COVID-19	Symptoms, signs, comorbidity, and mortality
Strang, P., Bergström J., and Lundström S. 2021, Sweden [36]	To examine symptom control data on all reported COVID-19-related deaths in hospitals and nursing homes, using the Swedish Register of Palliative Care (SRPC)	Descriptive national registry data study Data were collected 24/4–20 from the SRPC Follow-up period: 1/3–20 to 24/4–20	390 deaths of COVID-19: 253 deaths of COVID-19 in nursing homes 137 deaths of COVID-19 in hospitals	Symptoms, signs, and symptom relief
Strang, P., Martinsson L., Bergström J., and Lundström S. 2021, Sweden [37]	To compare symptom prevalence and relief in residents dying of COVID-19 in nursing homes with residents who were acutely referred to hospitals	National registry study Data were collected 24/8–20 from the Swedish Register of Palliative Care (SRPC) Follow-up period: 1/1–20 to 24/8–20	2105 deaths of COVID-19: 1903 deaths of COVID-19 in nursing homes 202 nursing home residents admitted to hospitals	Symptoms, signs, and symptom relief
Tang O., Bigelow B. F., Sheikh F., Peters M., Zenilman J. M., Bennett R. et al. 2020, USA [38]	To assess the association of symptom status and medical comorbidities on mortality and hospitalisation risk associated with COVID-19 in nursing home residents	Retrospective cohort study COVID-19 confirmed by RT-PCR test Follow-up period: 1/3–20 to 16/6–20	752 of 1970 nursing home residents with confirmed COVID-19	Symptoms, signs, comorbidity, and mortality
Tobolowsky, F. A., Bardossy A. C., Currie D. W, Schwartz N. G., Zacks R. L. T, Chow E. J. et al. 2021, USA [39]	To assess confirmed COVID-19 cases in a skilled nursing facility in the USA	Retrospective reviews of electronic health records COVID-19 confirmed by RT-PCR test Follow-up period: 28/2–20 to 16/3–20	101 of 118 nursing home residents with confirmed COVID-19	Symptoms, signs, comorbidity, hospitalisation, and mortality

^a^*RT-PCR test* real-time reverse-transcriptase polymerase chain reaction test; a diagnostic method for the detection of SARS-CoV-2 infection

### Data synthesis

Due to the heterogeneity in the study designs, methods, and outcomes in the articles, we presented the findings in a narrative synthesis. Data were grouped according to symptoms, signs, comorbidity, laboratory findings, medical treatment, and mortality among NH residents confirmed to have COVID-19. For single symptoms and findings comparable across studies, a weighted mean (*M*_weighted_, mean of proportions) and the range of proportions were calculated to account for the large variation in the number of participants in the studies.

### Ethical considerations

The articles examined in our systematic review were approved by ethics committees at the hospitals, universities or institutes, and local health authorities and/or institutional review boards.

This systematic review did not require ethical approval from the institution or an ethics committee due to secondary data analysis of existing data.

## Results

The 19 articles in our review, hereafter referred to as “studies”, were geographically spread across nine countries that included the United States (*n* = 9), Sweden (*n* = 2), the Netherlands (*n* = 2), and one each from Norway, Great Britain, Spain, Italy, France, and Belgium. All studies were published in 2020 or 2021 and conducted in the first and part of second wave of the pandemic before vaccinations were initiated. Table 2 describes the characteristics of each of the 19 studies. The total number of NH residents in the studies was 16,324, of whom 11,663 were confirmed to be positive for COVID-19 (*M*_weighted_ = 84.0%, range = 27.8%–100.0%). The number of residents in each study varied from 40 to 5256. Median age varied from 79 to 89 years, mean age from 75.2 to 88.8 years, and the age range was 51–107 years.

### Signs and symptoms

Fifteen of the studies [21–24, 27, 28, 30–35, 37–39] involved identification of signs and symptoms among NH residents confirmed to have COVID-19 as reported in Table 3. The most commonly identified symptoms and signs, reported as *M*_weighted_ and range, were fever (53.7%, 10.0%–86.0%), cough (56.5%, 19.6%–70.7%), hypoxia (32.3%, 3.0%–95.5%), delirium or confusion (31.2%, 3.0%–57.6%), dyspnoea, shortness of breath or respiratory distress (27.4%, 8.0%–61.0%), and/or abdominal pain, nausea, vomiting, and/or diarrhoea (24.1%, 6.0%–40.0%).

**Table 3:** Signs, symptoms, comorbidity, laboratory findings, medical treatment and mortality among nursing home (NH) residents confirmed to have COVID-19

**First author and year**	**Number of NH residents confirmed to have COVID-19**	**Age of participants in years in M (SD) or median (range)**	**Signs and symptoms** ***n* (%)**	**Comorbidity** ***n* (%)**	**Abnormal laboratory findings and chest X-rays**^a^ ***n* (%)**	**Medical treatment related to COVID 19 (not on regular basis)** ***n* (%)**	**Mortality in observation period, in weeks** ***n* (%) -**
Arons et al. (2020) [21]	57	78.6 (9.5)	*n* = 48 Fever: 5 (10) Cough: 16 (33) Shortness of breath: 4 (8) Confusion: 2 (4) Nausea: 3 (6) Diarrhoea: 2 (4) Sore throat: 3 (6) Malaise: 6 (13) Rhinorrhoea or congestion: 1(2) Dizziness: 2 (4) Headache: 1 (2) Asymptomatic at time of testing: 27 (56) Remained asymptomatic: 3 (6)	*n* = 48 Cognitive impairment: 28 (58) Cardiovascular disease: 39 (81) Diabetes: 18 (38) Chronic lung disease: 18 (38) Renal disease: 18 (38) Received haemodialysis: 3 (6) Cerebrovascular accident: 19 (40) Obesity: 11 (23)	Not informed	Not informed	*n* = 57 15 (26)—3
Atalla et al. (2021) [22]	111	Median: 87.0 (77.0–92.0)	*n* = 111 Fever: 89 (80.2) Cough: 48 (43.2) Shortness of breath: 16 (14.4) Respiratory distress: 26 (23.4) Hypoxemia (SpO2 < 94% in room air:106 (95.5) Confusion: 16 (14.4) Diarrhoea: 42 (37.8) Lethargy: 47 (42.3) Fatigue: 36 (32.4) Loss of appetite: 68 (61.3) Sore throat: 6 (5.4) Nasal congestion or rhinorrhoea: 7 (6.3) Altered mental state: 66 (59.5) Myalgia: 14 (12.6) Agitation or restlessness: 43 (38.7) Headache: 6 (5.4) Anxiety: 6 (5.4) Asymptomatic: 10 (9.0) Pre-symptomatic: 37 (33.3)	*n* = 111 Hypertension: 94 (84.7) Alzheimer’s disease or other dementias: 76 (68.5) Cardiovascular disease: 94 (84.7) Diabetes: 38 (34.2) Chronic pulmonary disease: 33 (29.7) Renal disease: 39 (35.1) History of malignancy: 7 (6.3) Obesity: 8 (7.2) Liver disease: 1 (0.9)	*n* = 87 High WBC count (> 10 k/μL): 7 (8) *n* = 82 Low Hb (< 12 g/dL): 47 (57) Low platelet count (< 150 k): 18 (22) High platelet count (> 400 k): 9 (11) *n* = 81 Low WBC count (< 4 k/μL): 13 (16) Low neutrophil count (< 2 k/μL): 13 (16) Elevated AST (> 40 U/L): 13 (16) *n* = 80 High neutrophil count (> 8 k/μL): 4 (5) Elevated ALP (> 146 U/L): 4 (5) *n* = 75 Low lymphocyte count (< 0.8 k/μL): 3 (4) Elevated ALT (> 40 U/L): 3 (4) *n* = 68 Elevated D-dimer (> 0.51 μg/mL): 54 (79) *n* = 48 Elevated PCT (> 0.15 ng/mL): 5 (10) *n* = 16 Elevated ferritin: 9 (56) *n* = 10 Elevated CRP (≥ 5 mg/L): 4 (40) *n* = 47 Abnormal chest X-ray: 27 (57)	*n* = 111 Supplemental oxygen or increased oxygen requirements: 49 (44.1) Anticoagulation: 70 (63.1) Enoxaparin: 53 (47.7) Rivaroxaban: 6 (5.4) Apixaban: 5 (4.5) Coumadin: 5 (4.5) Dabigatran: 1 (0.9)	*n* = 111 48 (43.2)—8
Beiting et al. (2021) [23]	172	75.4 (12.1)	*n* = 122 Fever > 99 °F: 79 (64.8) Cough: 27 (22.1) Shortness of breath: 14 (11.5) Hypoxia < 93% or change from baseline: 42 (34.4) Delirium: 60 (49.2) Diarrhoea: 8 (6.6) Nausea or vomiting: 7 (5.7) Tachycardia > 100 bpm: 21 (17.2) Fatigue: 48 (39.3) Anorexia: 61 (50.0) Sore throat: 5 (4.1) Myalgia: 6 (4.9) Hypotension < 100/60 or MAP < 65: 13 (10.7) Anosmia or ageusia: 1 (0.8) *n* = 172 COVID-19 positive and symptomatic: 103 (59.9) COVID-19 positive and pre-symptomatic: 19 (11.0) COVID-19 positive and asymptomatic: 50 (29.1)	*n* = 172 Hypertension: 157 (91.3) Cognitive impairment or dementia: 120 (69.8) Cardiac disease: 72 (41.9) Diabetes mellitus: 71 (41.3) Chronic pulmonary disease: 55 (32.0) Obesity (BMI > 30 kg/m^2^): 31 (18.0) Immunosuppressed: 17 (9.9)	*n* = 155 Leucocytosis (WBC > 10.8 1000/mm^3^): 13 (8.4) Leukopenia (WBC < 4.8 1000/mm^3^): 61 (39.4) Hypernatremia (Na > 145 mEq/L): 38 (24.5) AKI (Cr > 0.03 mg/dL or 1.5 × baseline): 46 (29.7) *n* = 147 AST > 22 U/L: 51 (34.7) *n* = 146 ALT > 35 U/L: 22 (15.1) *n* = 88 Abnormal chest radiograph: 27 (30.7)	*n* = 172 Oxygen supplementation: 47 (27.3) Intravenous fluids: 32 (18.6) Antibiotics: 37 (21.5) Exposure to novel therapeutics: 7 (4.1) (e.g. steroids and hydroxychloroquine): 7 (4.1)	*n* = 172 27 (15.7)—4
Bielza et al. (2021) [24]	341 (in NH)	Median: 88 (83.5—92.5)	*n* = 341 Fever: 147 (43.1) Cough: 67 (19.6) Dyspnoea: 186 (54.5) Abdominal pain: 1 (0.3) Diarrhoea: 11 (3.2) Vomiting: 8 (2.3) Symptoms of upper respiratory tract: 10 (2.9) Epileptic seizures: 4 (1.2) Chest pain: 3 (0.9) Arthromyalgia: 2 (0.6) Headache: 3 (0.9) Anosmia: 2 (0.6) Severe case: 253 (74.2)	*n* = 341 Hypertension: 206 (60.4) Dementia: 197 (57.8) Heart failure: 34 (10.0) Ischemic heart disease: 32 (9.4) Diabetes: 52 (15.2) COPD: 25 (7.3) Chronic renal disease: 30 (8.8) Cirrhosis: 1 (0.3) Obesity: 20 (5.9) Chronic neurological disease: 76 (22.3) Chronic inflammatory disease: 6 (1.8) Solid neoplasm: 30 (8.8) Haematological neoplasm: 4 (1.2) Sleep apnoea syndrome: 7 (2.1)	Not informed	*n* = 341 Antibiotic: 199 (58.4) Fluid therapy: 168 (49.3) Hydroxychloroquine: 11 (3.2) Enoxaparin: 256 (75.1) Inhalers: 259 (76.0)	*n* = 341 158 (46.3)—4
Brouns et al. (2020) [25]	67	84.4 (8.5)	Not informed	*n* = 67 Hypertension: 33 (49.3) Dementia: 45 (67.2) Myocardial infarction: 7 (10.4) Congestive heart failure: 13 (19.4) Diabetes mellitus, type 2: 9 (13.4) Cerebrovascular accident or transient ischaemic attack: 20 (29.9)	Not informed	Not informed	*n* = 67 35 (52.2)—6
De Spiegeleer et al. (2020) [26]	154	85.9 (7.2)	*n* = 154 Asymptomatic during study period: 41 (27) Serious COVID-19: 37 (24.0)	*n* = 154 Hypertension: 39 (25.3) Diabetes mellitus: 28 (18.2)	Not informed	Not informed	Not informed
Graham et al. (2020) [27]	126	Median 83	*n* = 126 Fever: 30 (23.8) Cough or breathlessness: 41 (32.5) Confusion or altered behaviour: 43 (34.1) Diarrhoea or vomiting: 2 (1.6) Anorexia: 34 (27.0) Asymptomatic: 54 (42.9)	Not informed	Not informed	Not informed	*n* = 126 21 (16.7)—8
Kittang et al. (2020) [28]	40	86.2	*n* = 40 Fever: 9 (23) Cough: 17 (43) Dyspnoea: 19 (48) Acute confusion and/or change in behaviour: 20 (50) Acute gastrointestinal symptoms: 16 (40) Marked reduction in general condition: 34 (85) New or increased risk of falling: 13 (33) Muscular pain, sore throat, acute urinary incontinence or headache: 8 (20) Asymptomatic at first test: 9 (27)	*n* = 40 Hypertension: 21 (53) Cognitive impairment: 33 (83) Chronic heart disease: 16 (40) Diabetes: 12 (30) Chronic pulmonary disease: 10 (25) Chronic renal failure: 9 (23) Previous or current smoker: 13 (33) BMI > 30 or < 18: 11 (28) Cancer: 9 (23) ≥ 3 comorbid conditions: 35 (88) ≥ 2 comorbid conditions: 38 (95)	Not informed	Not informed	*n* = 40 21 (53)—5
Lally et al. (2021) [29]	775	75.6 (10.8)	Not informed	*n* = 775 Dementia: 537 (69.3) Diabetes: 308 (39.7) Pulmonary disease: 264 (34.1) Renal disease: 195 (25.2) Obesity: 118 (15.2) Hypothyroid: 91 (11.7) Tumour: 131 (16.9) Weight loss: 138 (17.8) Alcohol use disorder: 96 (12.4) Drugs use disorder: 60 (7.7) Any substance use disorder: 125 (16.1) Psychiatric diagnosis: 541 (69.8) Psychoses: 327 (42.2) Depression: 330 (42.6)	Not informed	Not informed	*n* = 775 160 (20.6)—4
Panagiotou et al. (2021) [30]	5256	Median: 79 (69—88)	*n* = 5256 Fever: 2654 (50) Shortness of breath: 582 (11) Hypoxia: 979 (19) Tachycardia: 900 (17)	*n* = 5256 Hypertension: 4116 (78) Dementia: 2503 (48) Cognitive impairment: - Mild: 1179 (22) - Moderate: 1547 (29) - Severe: 463 (9) Heart failure: 1201 (23) Coronary artery disease: 1227 (23) Type 2 diabetes: 2112 (40) Asthma or COPD: 1366 (26) Chronic kidney disease: 1385 (26)	Not informed	Not informed	*n* = 5256 1122 (21.3)—4
Patel et al. (2020) [31]	35	Median: 82 (75—92)	*n* = 35 Fever: 15 (43) Cough: 9 (26) Shortness of breath: 5 (14) Hypoxia: 1 (3) Loss of consciousness or delirium: 1 (3) Fatigue: 9 (26) Loss of appetite: 4 (11) Sore throat: 3 (9) Myalgia: 2 (6) Chills: 1 (3) Seizures: 1 (3) Asymptomatic during follow-up: 13 (37) *n* = 33 Asymptomatic at the time of testing: 14 (42)	Not informed	Not informed	Not informed	*n* = 35 10 (28.6)—4
Poloni et al. (2020) [32]	57	82.8 (6.8)	*n* = 57 Fever or other typical symptoms: 49 (86.0) Delirium: 21 (36.8) Asymptomatic throughout the clinical course: 8 (14.0)	*n* = 57 Hypertension: 31 (54.4) Dementia: 57 (100.0) Cardiovascular disease: 18 (31.6) Diabetes mellitus: 11 (19.3) Chronic pulmonary disease: 8 (14.0) Malignancy: 6 (10.5)	*n* = 57 Lymphocyte count (1.5 – 4 × 10^9^/L) 50/57 (89.5): 1.3 (1.1–1.6) CRP (2.9 – 76.2 nmol/L) Overall: 50/57 (87.7): 152.4 (29.5–361.9) Living group: 88.6 (29.5–269.5) Deceased group: 367.6 (196.2–740.0)	Not informed	*n* = 57 14 (24.6)—3
Rutten et al. (2020) [33]	1538	84 (8.7)	*n* = 1455 Fever: 917 (63) *n* = 1431 Cough: 900 (63) *n* = 1373 Shortness of breath: 417 (30) *n* = 1023 Decreased oxygen saturation: 453 (44) *n* = 1288 Delirium, confusion or drowsiness: 372 (29) *n* = 976 Sore throat: 94 (10) *n* = 417 Nausea or vomiting: 48 (12) Fatigue: 93 (22) Diarrhoea: 74 (18) Malaise: 73 (18) Rhinorrhoea: 52 (12) Common cold: 52 (12)	*n* = 1525 Dementia: 945 (62) Cardiovascular disease: 763 (50) Cerebrovascular disease: 625 (41) Diabetes mellitus: 397 (26) Chronic respiratory disease: 275 (18) Reduced kidney function: 275 (18) Parkinson disease: 92 (6)	Not informed	Not informed	*n* = 1538 646 (42.0)—4
Sacco et al. (2020) [34]	41	88.8 (7.0)	*n* = 41 Temperature changes: 28 (68) - Normothermia: 13 (32) - Hypothermia < 36 °C: 4 (10) - Hyperthermia > 38 °C: 24 (59) Cough: 20 (49) Dyspnoea: 25 (61) Polypnea: 17 (42) - Between 23 and 29/minute: 8 (20) - ≥ 30/minute: 9 (22) Pulse oximetry under 90%: 19 (46) Delirium (over- or hypoactive): 3 (7) Diarrhoea: 5 (12) Nausea: 2 (5) Vomiting: 1 (2) Asthenia: 14 (34) Anorexia: 7 (17) Myalgia or arthralgia: 3 (7) Low blood pressure: 2 (5) Fall: 6 (15) Altered consciousness: 4 (10) Rhinitis: 8 (20) Odynophagia: 2 (5) Anosmia: 1 (2) Conjunctivitis: 1 (2) Other signs^b^: 9 (22)	Not informed	Not informed	Not informed	*n* = 41 11 (27.0)—6
Shi et al. (2020) [35]	146	85.0 (9.3)	*n* = 146 Symptoms at time of testing: Fever: 31 (21.4) Cough: 35 (24.1) Delirium: 27 (18.6) Vomiting: 13 (9.0) Diarrhoea: 9 (6.2) Anorexia: 26 (17.9) Asymptomatic: 66 (45.5) Symptoms at any time: Delirium: 83 (57.6) Anorexia: 102 (70.8) Fall: 22 (15.1) Days of fever (M ± SD): 1.8 ± 2.0 (max. 9 days)	*n* = 146 Cognitive impairment - None or mild: 46 (33.8) - Moderate: 65 (47.8) - Severe: 25 (18.4) Congestive heart failure: 12 (8.6) Diabetes: 25 (18.0) COPD: 12 (8.6))	Not informed	*n* = 146 Required oxygen: 58 (40.0)	*n* = 146 44 (30.1)—7
Strang, Bergström et al. (2021)^c^ [36]	253	*M* = 86.6 (range: 63 -104)	Not applicable	Not informed	Not informed	p.r.n. prescriptions (*n* = 253)^d^ Strong opioid: 243 (96) Tranquiliser: 242 (96) Antiemetic: 238 (94) Antimuscarinic: 243 (96)	Not applicable
Strang, Martinsson et al. (2021)^e^ [37]	1903	*M* = 86.7 (range: 57 -107)	*n* = 1811 Breathlessness: 556 (31) *n* = 1676 Delirium: 423 (25) *n* = 1797 Anxiety: 1015 (56) *n* = 1867 Respiratory secretions: 956 (51)	Not informed	Not informed	*n* = 1889 Parenteral or enteral fluids, nutrition during on last day of life: 116 (6)	*n* = 1903 1903 (100.0)—34
Tang et al. 2020 [38]	752	Asymptomatic: 74.7 Symptomatic: 76.5	*n* = 328 Fever: 161 (49.1) Cough: 195 (59.5) Shortness of breath: 69 (21.0) Vomiting: 14 (4.3) Diarrhoea: 23 (7.0) Sore throat: 7 (2.1) Muscle aches: 28 (8.5) Runny nose: 8 (2.4) Nasal congestion: 12 (3.7) Chest congestion: 24 (7.3) Chills: 2 (0.6) Shaking: 1 (0.3) *n* = 752 Asymptomatic at time of testing: 424 (56.4) Symptomatic at time of testing: 328 (43.6)	*n* = 752 Hypertension: 664 (88.3) Dementia: 378 (50.3) Coronary heart disease: 240 (31.9) Heart failure: 198 (26.3) Diabetes: 380 (50.5) Asthma: 54 (7.2) COPD or emphysema: 146 (19.4) Chronic kidney disease, stage 1–5: 200 (26.6) End-stage kidney disease: 90 (12.0) Cancer: 61 (8.1) Atrial fibrillation: 146 (19.4) Peripheral vascular disease: 168 (22.3) DVT or PE: 107 (14.2) Anaemia: 389 (51.7) Hypothyroidism: 123 (16.3) HIV: 10 (1.3) Chronic hepatitis C: 21 (2.8) Cerebrovascular disease: 277 (36.8) Depression: 421 (56.0)	Not informed	Not informed	*n* = 242 155 (64) – 15
Tobolowsky, et al. (2021) [39]	101	Median: 83 (51–100)	*n* = 82 Fever (≥ 100.0 °F): 40 (49) Low-grade fever (99.0–-99.9 °F): 24 (29) Cough: 58 (71) Shortness of breath: 29 (35) Tachypnoea: 42 (51) Change in oxygen status: 45 (55) Change in mental state: 24 (29) Nausea or vomiting: 8 (10) Acute diarrhoea: 7 (9) Tachycardia: 50 (61) Sputum production: 26 (32) Fatigue: 19 (23) Lethargy: 15 (18) Rhinorrhoea or nasal congestion: 5 (6) Conjunctivitis: 4 (5) Chills: 1 (1) Headache: 1 (1) Sore throat: 4 (5) Myalgia: 1 (1) *n* = 101 Asymptomatic: 4 (4) Pre-symptomatic: 12 (11.9)	*n* = 101 Hypertension: (81) Dementia: (46) Cardiac disease: (65) Diabetes mellitus: (33) Pulmonary disease: (28) Renal disease: (42) Hepatic disease: (8) Malignancy: (17) Immunodeficiency: (1) Obesity: (33) Neurologic disease: (38)	Not informed	Not informed	*n* = 101 35 (34.7)—4

*AKI* Acute kidney injury, *ALP* Alkaline phosphatase, *ALT* Alanine aminotransferase, *AST* Aspartate aminotransferase, *COPD* Chronic obstructive pulmonary disorder, *CRP* C-reactive protein, *Hb* Haemoglobin, *PCT* Procalcitonin, *WBC* White blood cells

^a^Abnormal chest X-rays described in Atalla et al. (2021) [22] included interstitial densities (8/27, 29.6%), unilateral base infiltrates (7/27, 29.6%), patchy unilateral densities (5/27, 18.5%) and patchy bilateral densities (4/27, 14.8%)

^b^Sacco et al. (2020) [34] observed other signs, including dizziness (*n* = 3), headache (*n* = 2), facial erythrosis (*n* = 2), pallor, erythematous rash, marble skin, chest pain, crying (*n* = 1 each)

^c, e^Strang, Martinsson et al. (2021) [37] described data only from deaths of COVID-19, and, for that reason, they reported 100% mortality. Due to overlapping of data found in the studies by Strang, Bergström et al. (2021) [36] and Strang, Martinsson et al. (2021) [37], figures for mortality and symptoms/signs represent the study that observed the longest period

^d^In Strang, Bergström et al. (2021) [36], “p.r.n. prescriptions” means “pro re nata prescriptions”, or medicine taken not on regular basis but as needed

Among the studies that did not identify any symptoms in COVID-19-positive NH residents at the time of testing, the *M*_weighted_ was 50.1% (3.0%–56.4%). For the studies that identified an asymptomatic course during the follow-up period, the *M*_weighted_ was 23.5% (4.0%–37.1%).

Two studies, Bielza et al. and De Spiegeleer et al. [24, 26] revealed that some NH residents displayed a severe course of COVID-19; 253 of 341 (74.2%) and 37 of 154 (24%) of the NH residents, respectively. Although De Spiegeleer et al. did not define the symptoms of severe courses of COVID-19 in their study, Bielza et al. defined such courses as involving a temperature exceeding 38 °C, systolic blood pressure less than 100 mm Hg, a heart rate exceeding 100 beats per minute, basal oxygen saturation of less than 90%, respiratory rate exceeding 30 per minute, and an altered level of consciousness.

### Comorbidities

Comorbid conditions in NH residents, as shown in Table 3, were identified in 16 studies [21–30, 32–35, 38, 39]. In the studies of Graham et al. and Sacco et al., comorbidities were not divided according to whether the patients were positive or negative for COVID-19; thus, their data were omitted from our study. The remaining 14 studies identified comorbid conditions in NH residents confirmed to have COVID-19. The most frequent comorbidities reported and expressed as *M*_weighted_ and range were hypertension (78.6%, 25.3%–91.3%), dementia or cognitive impairment (55.3%, 46.0%–100.0%), cardiovascular disease (52.0%, 8.2%–84.7%), diabetes (38.7%, 13.4%–50.5%), renal disease (27.1%, 8.8%–42.0%), and pulmonary disease (26.0%, 7.3%–38.0%).

### Laboratory findings and chest X-rays

Laboratory findings for NH residents with confirmed COVID-19 are reported in three of the articles [22, 23, 32] as described in Table 3.

Atalla et al. found elevated C-reactive protein levels (CRP ≥ 5 mg/L) in four of 10 (40%) NH residents or whom laboratory tests were performed [22]. By comparison, Poloni et al. found that 50 of 57 (87.7%) NH residents had an elevated CRP (reference range 2.9–76.2 nmol/L) with an overall median of 152.4 nmol/L. In their study, CRP was higher in the deceased group (367.6 nmol/L) than in the living group (88.6 nmol/L) [32].

Atalla et al. detected a low lymphocyte count (< 0.8 k/μL) in three of 75 (4%) NH residents, whereas Poloni et al. found that 51 of 57 (89.5%) NH residents had low lymphocyte counts with a median count of 1.3 × 10^9^/L [22, 32].

Both Atalla et al. and Beiting et al. assessed low and high white blood cell (WBC) counts. Atalla et al. found a low WBC count (< 4 k/μL) in 13 of 81 (16%) NH residents, whereas Beiting et al. found that 61 of 155 (39.4%) NH residents displayed a low WBC count (< 4.8 1000/mm^3^). In the two studies, high WBC counts (> 10 k/μL and > 10.8 1000/mm^3^, respectively) were found in seven of 87 (8%) and in 13 of 155 (8.4%) NH residents, respectively [22, 23].

Regarding elevated D-dimer levels (> 0.51 μg/mL), Atalla et al. found such levels in 54 of 90 (60.0%) NH residents tested, and 47 of the 90 (52%) had low haemoglobin (Hb < 12 g/dL) [22]. In Beiting et al.’s study, 46 of 155 (29.7%) NH residents had acute kidney injury indicated by elevated creatinine levels (> 0.03 mg/dL or 1.5 × baseline), and 38 of the 155 (24.5%) exhibited hypernatremia (Na > 145 mEq/L) [23].

Those two research groups also reported abnormal chest X-ray results in their studies [22, 23]. In Atalla et al.’s study, the most common findings were interstitial densities (8/27 residents, 29.6%), unilateral base infiltrates (7/27, 29.6%), patchy unilateral densities (5/27, 18.5%), and patchy bilateral densities (4/27, 14.8%) [22].

### Medical treatment

The medical and pharmacological treatments of the NH residents confirmed to have COVID-19 are described in Table 3. The treatments were administered for COVID-19 and did not include treatment given on a regular basis for other conditions. Six studies presented data on medical and pharmacological treatments [22–24, 35–37]. The treatments were used to improve outcomes, as part of palliative care, or as end-of-life treatment. Treatments included inhalers, antibiotics, anticoagulation, oxygen supplementation, parenteral or enteral fluids or nutrition, hydroxychloroquine, and medication based on the NH resident’s needs (such as pro re nata prescriptions). In the studies of Beiting et al. and Bielza et al., treatment with antibiotics were described. This treatment was prescribed for 21.5% and 58.4% of the NH residents, respectively [23, 24]. Oxygen supplementation was described in the studies of Atalla et al., Beiting et al., and Shi et al. In these studies, 44.1%, 27.3%, and 39.7% of the NH residents in the studies received supplemental oxygen [22, 23, 35]. Two of the studies by Atalla et al. and Bielza et al. described treatment with the anticoagulation agent, Enoxaparin. In the study of Atalla et al. 47.7% received this treatment, and in the study of Bielza 75.1% [22, 24]. Posology was not stated.

### Transfer to hospital

Transfers to hospital were reported in six of the included studies, and the rate of hospital transfers varied from 6.9% to 50% for residents with confirmed COVID-19 [21, 23, 24, 31, 37, 39]. Strang et al. reported that nursing home residents who were transferred to hospital were generally younger (83.3 years versus 86.7 years; *p* < 0.00001) and more often male (*p* < 0.0001) [37]. The study of Tobolowsky et al. showed that relative risk (RR) of hospitalisation was higher among COVID-19- positive NH residents with underlying hepatic disease (RR 1.6, 95% confidence interval [CI] 1.1–2.2) or obesity (RR 1.5, 95% CI 1.1–2.1) and for those treated with continuous positive airway pressure (RR 1.6, 95% CI 1.2–2.1) [39].

### Mortality

Mortality in NH residents with confirmed COVID-19 was reported in 17 of the studies as shown in Table 3 [21–25, 27–35, 37–39]. The reported mortality rates ranged from 15.7% to 100.0% while the observation periods varied from three weeks to eight months. Cognitive impairment or dementia, hypoxia or change in oxygen status, old age (≥ 84 years), and male gender were associated with higher mortality in 10 studies [22, 24, 25, 27, 30, 32, 33, 36, 37, 39]. Overall, 11,008 NH residents confirmed to have COVID-19 were included in the 17 studies reporting mortality, and 4427 (40.2%) of them died during the observation period of the studies.

## Discussion

The aim of our systematic review was to describe the clinical expressions, characteristics, and treatments of NH residents with confirmed COVID-19. Fever, respiratory issues, and delirium or confusion were most frequently reported signs and symptoms. Not having symptoms at the time of testing or during the follow-up period was also common. Moreover, some NH residents displayed a severe course of COVID-19. The most frequently reported comorbidities among NH residents who tested positive for COVID-19 were hypertension, dementia or cognitive impairment and cardiovascular disease. Few articles reported laboratory findings or medical treatments, whether those treatments were used to improve outcomes, as part of palliative care, or as end-of-life treatment. Mortality rates due to COVID-19 were high, and dementia or cognitive impairment, changes in oxygen status, old age, and male gender may be associated with higher mortality.

### Clinical presentation

Regarding symptoms and signs of COVID-19, approximately half of the NH residents in the studies had fever and cough while one-third had hypoxia. Those findings align with the findings of other studies reporting these signs and symptoms in both NH residents and older adult patients in hospitals [12, 17, 40, 41]. Several of the NH residents with confirmed COVID-19 had no symptoms at the time of testing but developed one or more symptoms compatible with COVID-19 during the course of the disease. Our findings also show that an asymptomatic course during the follow-up period may occur. It should not be overlooked that those who were defined as asymptomatic may have had symptoms weeks before testing, but is it likely that some of the NH residents underwent an asymptomatic course. Thus, although NH residents with COVID-19 may present with typical symptoms and signs such as fever, cough, and dyspnoea, these symptoms may also be absent in this population. COVID-19’s capacity to present without symptoms or follow an asymptomatic course has also been described for the general population [2]. A systematic review and meta-analysis estimated that 42.8% of the general population confirmed to have COVID-19 had no symptoms at the time of testing, and 35.1% remained asymptomatic during the follow-up period. This study also revealed that younger people and children had a higher percentage of asymptomatic courses than older adults. People with no comorbidities also had a higher percentage of asymptomatic courses than for those with comorbidities [42]. Due to advanced age and frailty, the basal body temperature decreases, and, for that reason, it has been suggested that fever in NH residents should be defined as a single oral temperature exceeding 37.8 °C (> 100 °F), repeated oral temperatures exceeding 37.2 °C (> 99 °F), or those exceeding 1.1 °C (> 2 °F) over the basal temperature [15, 16].

Other atypical signs or symptoms indicating infection in NH residents could be a change in mental or cognitive state such as delirium or confusion [15, 43]. Our systematic review revealed that among the studies reporting delirium or confusion, an average of 31.2% (*M*_weighted_) of the NH residents was affected by the condition as a component of COVID-19, which is also described by Hashan et al. [17]. NH residents have a higher risk of developing delirium due to their higher rates of dementia, multimorbidity, frailty and older age than other populations [43]. Delirium is characterised by the acute appearance of symptoms, such as confusion, disorientation, delusions, hallucinations, agitation, hyperactivity, or hypoactivity [43, 44]. The condition can also have serious negative consequences, including the development or worsening of dementia in addition to an increased risk of hospitalisation and mortality. NH residents often have more frequent worsening patterns of delirium than patients in hospital, and the hypoactive form of delirium may be especially severe for people with dementia [43]. It is therefore imperative to highlight that delirium can be a presentation of COVID-19 among NH residents due to the severe negative consequences of this condition.

In our systematic review, changes in oxygen status were associated with higher mortality and thus represent a sign that is important to recognise among NH residents. Among patients admitted to hospital due to COVID-19, both dyspnoea and low oxygen saturation were associated with mortality [45]. The high mortality rate presented in this systematic review must be interpreted with caution. However, a report on mortality among nursing home residents based on 22 countries shows a similar result with an average of 41% [13]. Based on our results, it is not possible to determine any differences in the impact caused by COVID-19 on short- or long-term mortality.

Identifying the symptoms of COVID-19 in NH residents is important for early diagnosis and proper treatment before the condition worsens in addition to reducing the risk of the further transmission of SARS-CoV-2. In the case of suspected infection, having a liberal testing system to confirm or rule out COVID-19 can help to prevent the undetected spread of the virus. Due to the high percentage of asymptomatic patients, relying solely on symptom-based screening is insufficient for reliably identifying COVID-19. The presence of symptoms, such as fever and cough, should prompt further testing [46]. Future research should also investigate how the assessment of symptoms and the clinical evaluation of COVID-19 in NH residents are performed by NH staff.

The results of abnormal laboratory findings reported in the articles indicate elevated CRP and D-dimer levels, lymphopenia, leukopenia, and acute kidney injury among NH residents confirmed to have COVID-19. However, given the scarcity of studies that have reported such findings, it remains difficult to draw conclusions about the frequency of those findings and the significance of the test results. A meta-analysis among older adult patients with COVID-19 has shown that lymphopenia and leukopenia may be common in that population and that acute kidney injury is a common complication [41]. Another meta-analysis has shown that acute kidney injury and elevated CRP and/or D-dimer levels may be clinically relevant to mortality among patients with COVID-19 who are admitted to hospital [45]. Even though these findings may not be directly transferable to the NH resident population, who may differ in age and disease burden from patients admitted to hospital, it is important to note these results, since our systematic review indicate similar laboratory responses. The abnormal X-ray results considered in our systematic review were also too few to be generalisable; however, other studies have shown that pulmonary infiltrates among older adults with COVID-19 admitted to hospital may be common [40, 41, 47].

### Characteristics of residents with COVID-19

In general, frequently reported diagnoses among NH residents are hypertension, cardiovascular disease, dementia, diabetes, and chronic obstructive pulmonary disorder, and studies also show that NH residents usually display three or more underlying health conditions [7, 9]. In our systematic review, we found that a high proportion of NH residents confirmed to have COVID-19 had hypertension, and more than half had dementia or cognitive impairment and cardiovascular disease. Based on our findings, we cannot confirm whether a higher incidence of multimorbidity among COVID-19 positive NH residents than those NH residents without COVID-19 exist. Dementia or cognitive impairment was associated with a higher risk of mortality, and studies show that people with dementia have a higher risk of getting COVID-19 and that their rate of mortality due to COVID-19 is greater than for people without dementia [48, 49]. NH residents are usually multimorbid adults who are at least 80 years old, and both multimorbidity and old age lead to an increase in the risk of a severe course of COVID-19 and mortality [7, 8, 10, 11] as do hypertension and cardiovascular disease, especially among NH residents [6, 12]. In the literature analysed in our systematic review, the specific description of severe courses of COVID-19 is not frequently outlined. Bielza et al. [24] described severe courses as involving a temperature exceeding 38 °C, systolic blood pressure less than 100 mm Hg, a heart rate exceeding 100 beats per minute, basal oxygen saturation of less than 90%, a respiratory rate exceeding 30 per minute, and an altered level of consciousness. Although residents with those signs were cared for in their NHs [24], it remains debated whether NH residents should be transferred to hospital or treated in their respective NHs for COVID-19. Few of the included studies reported transfers to hospital for NH residents with confirmed COVID-19, and the findings in our systematic review are too scarce to conclude anything about risk factors for hospitalisation for this population. However, most guidelines stipulate that NH residents should receive treatment and care in their NHs when possible [50]. In general, NHs have faced tremendous challenges in recruiting enough staff who are sufficiently qualified, a trend dating back to before the pandemic. The first wave of the pandemic greatly exacerbated those problems as several members of staff had to be absent due to restrictions. Added to that, some managers had to work from their own homes, there were quarantine requirements, physicians were absent and only available via telephone or video, and more staff were needed to meet the residents’ needs for more comprehensive care [51]. Hospitals generally have more resources and larger staffs of physicians, nurses, and other relevant staff members in addition to access to on-site services, such as laboratory- and X-ray departments [14]. Those differences between hospitals and NHs are relevant when deciding whether to transfer an NH resident to a hospital or not. From another point-of-view, NHs are both the homes and health institutions for their residents. When the pandemic broke out, it was exceptionally demanding for NH employees to manage the intensified care needs of their residents brought about by COVID-19 [51]. Determining whether a NH resident should be admitted to hospital may also be partly based on the resident’s preferences in relation to treatment. The benefits and risks of transferring NH residents to hospital should also be considered. After all, the right to adequate healthcare is a human right regardless of age, functional state, or place of residence [50, 52].

### Interventions

The medical and pharmacological treatments of NH residents confirmed to have COVID-19 identified in our systematic review were used to improve outcomes, as part of palliative care, or as end-of-life treatment. The treatments described were administered early on during the pandemic, and hydroxychloroquine was used in some severe cases of COVID-19. It turned out, however, that hydroxychloroquine did not provide any benefits for patients with COVID-19. In December 2020, it was strongly advised not to use the drug [53]. NH residents who displayed a severe course and/or who were near the end of life received oxygen therapy, parenteral or enteral fluids, and pro re nata prescriptions of palliative drugs. Despite those findings and to our knowledge studies that have described the medical and pharmacological treatment received by NH residents confirmed to have COVID-19 are scarce. Medical and pharmacological treatment require the close follow-up of patients; however, the staffing situation in NHs has continued to be strained [51, 52]. It is therefore possible that not all NHs provide equal opportunities for medical and pharmacological treatment, especially not treatment for the consequences of COVID-19. In the future, researchers should therefore focus on how NH staff offer treatment, care, and nursing to NH residents with severe courses of COVID-19.

### Strengths and limitations

In this review we rigorously followed both the Preferred Reporting Items for Systematic reviews and Meta-Analyses (PRISMA) 2020 statement and a systematic qualitative assessment system of the included studies, the Newcastle–Ottawa assessment scale (NOS). We conducted thorough literature searches using relevant databases (PubMed, CINAHL, AgeLine, Embase and PsycINFO), but the risk of missing studies cannot be ignored. The included studies were conducted exclusively in Europe or North America, possibly due to the geographical spread of the pandemic in which it first broke out and the exclusion of studies not published in English. In addition, the studies varied in sample size, length of follow-up period, and acquisition of data. Given such heterogeneity in study designs, methods, and outcomes, it proved impossible to perform a meta-analysis along with our systematic review. Our findings must therefore be interpreted with caution based on these limitations. Nevertheless, our review has allowed us to summarise important clinical findings about COVID-19 pertaining to this vulnerable population of NH residents.

## Conclusions

Our systematic review has revealed that NH residents have several risk factors for severe illness and death caused by COVID-19. In this population, COVID-19 can appear with both typical and atypical symptoms but can also be asymptomatic. Delirium can be an atypical symptom of COVID-19 among NH residents, one that warrants close attention given the severe negative consequences of the condition. Using a liberal testing system to confirm or rule out COVID-19 suspected in NHs can prevent severe courses of the disease and death due to the undetected spread of the virus. Considering that NH residents with COVID-19 preferably should be treated at their NHs, having enough high-quality staff at the facilities is vital, especially because the virus continually emerges with new variants. Future research thus needs to examine how symptoms are assessed and how COVID-19 is clinically evaluated among NH residents by NH staff. Beyond that, the treatment and care of NH residents with severe courses of COVID-19 need to be investigated in greater depth.

## Data Availability

The authors declare that all data generated or analysed during this study are included in this published article (and its supplementary information files).
